# Effects of Cinnamon (*Cinnamomum* spp.) in Dentistry: A Review

**DOI:** 10.3390/molecules25184184

**Published:** 2020-09-12

**Authors:** Spartak Yanakiev

**Affiliations:** Medical College Y. Filaretova, Medical University—Sofia, Yordanka Filaretova Street 3, 1000 Sofia, Bulgaria; s.yanakiev@mc.mu-sofia.bg; Tel.: +35-98-8644-5108

**Keywords:** cinnamon essential oil, dentistry, oral pathogens, oral biofilm, candida, antimicrobial effect, dental caries, endopathogens, cinnamaldehyde, eugenol

## Abstract

Dental medicine is one of the fields of medicine where the most common pathologies are of bacterial and fungal origins. This review is mainly focused on the antimicrobial effects of cinnamon essential oil (EO), cinnamon extracts, and pure compounds against different oral pathogens and the oral biofilm and the possible effects on soft mouth tissue. Basic information is provided about cinnamon, as is a review of its antimicrobial properties against the most common microorganisms causing dental caries, endodontic and periodontal lesions, and candidiasis. Cinnamon EO, cinnamon extracts, and pure compounds show significant antimicrobial activities against oral pathogens and could be beneficial in caries and periodontal disease prevention, endodontics, and candidiasis treatment.

## 1. Introduction

Dental medicine is one of the fields of medicine where the most common pathologies are of bacterial and fungal origins. Widely spread diseases like dental caries, periodontal disease, and endodontic lesions are caused by well-known bacterial and fungal pathogens: *Streptococcus mutans, Streptococcus salivarius, Streptococcus sanguinis, Porfiromonas gingivalis, Prevotella intermedia, Actinobacilus actinomycetemcomitans, Enterococcus faecalis, Candida albicans,* etc. [1]. Preventive medicine relies mostly upon reducing the bacterial biofilm via oral hygiene. The most often used active ingredients in mouth rinses and toothpastes are chlorhexidine, hyaluronic acid, and fluorides. Although effective, chemical products may have some clinical disadvantages: teeth discoloration, taste alterations, mouth dryness, supragingival calculus accumulation, and oral mucosal lesions [2,3,4].

The attention of many researchers has focused on the antimicrobial properties of traditional medical substances, like essential oils (EOs) [5,6,7]. EOs and extracts have demonstrated effective antibacterial and antifungal properties [8,9,10,11]. In the field of dental medicine, oral hygiene products based on herbal extracts are well-known [12,13,14]. One of the substances most used by dental professionals is eugenol, which is an active component in root canal sealers, cements, and others. One of the EOs subjected to investigation in dentistry is cinnamon (*Cinnamomum* spp., Lauraceae family) [15].

Cinnamon is a widely known culinary herb and traditionally used in medicine applications. The effect of cinnamon has been studied during pregnancy [16], for diabetes control [17], and gynecological problems [18]. Its anti-inflammatory, cardioprotective, antioxidative, and antimicrobial properties have also been researched [19]. Thus, cinnamon EO, cinnamon extracts, and pure compounds, due to their antibacterial, antifungal, and other properties, have potential uses in mouth rinses, toothpastes, or as a root canal irrigant, showing promise as an antimicrobial agent in dentistry.

This review focused mainly on the antimicrobial effects of cinnamon EO, extracts, and pure compounds against different oral pathogens and the oral biofilm and the possible effects on mouth tissues.

## 2. Basic Characteristics of Cinnamon and Its Chemical Composition

Cinnamon (*Cinnamomum* spp., Lauraceae family) includes more than 250 evergreen trees spread mainly in Asia, China, and Australia [20]. Many species have been studied in the literature, and some of them in the field of dental medicine. Two of the most studied types of cinnamon are *Cinnamomum verum* or *Cinnamomum zeylanicum* (true cinnamon, Ceylon cinnamon, or Mexican cinnamon). *C. verum*’s older botanical name, *C. zeylanicum*, derives from Sri Lanka’s older name, Ceylon. Well-studied is also *Cinnamomum aromaticum* or *Cinnamomum cassia* (Cassia cinnamon or Chinese cinnamon). The other two main species of cinnamon are *Cinnamomum burmannii* (also called Korintje, Java, or Indonesian cinnamon) and *Cinnamomum loureiroi* (Vietnamese or Saigon cinnamon) [19,21,22].

EOs and extracts have been isolated from the different parts of cinnamon, such as the leaves, bark, fruits, root bark, flowers, and buds. More than 80 compounds have been identified, and the compositions vary due to many factors [20]. The main components of cinnamon EOs and extracts are cinnamaldehyde, eugenol, phenol, and linalool. Cinnamon bark EO has a higher cinnamaldehyde content (65–80%) and a low eugenol content (5–10%). The extract from leaves is rich in eugenol (10–95%). Roots are rich in camphor [23,24,25]. The leaf extracts may also have a high cinnamaldehyde content [26]. Table 1 presents the most abundant compounds found in different cinnamon species and parts of the plant.

*C. zeylanicum* and *C. cassia* are the most-studied types of cinnamon. Doh et al. reported that, based on the analysis of phylogenetic relationships, *C. cassia* and *C. zeylanicum* are clustered in different groups [45]. One of the main differences between *C. zeylanicum* and *C. cassia* is the coumarin content. *C. cassia* tends to have a higher percentage of coumarin, thus posing more health risks due to the anticoagulant, cancerogenic, and hepatotoxic properties of coumarin [46]. Jose et al. reported a higher coumarin content in the leaves of *C. cassia* (481–2462 mg/kg) in comparison to the bark (3–86 mg/kg) [31]. In comparison, the coumarin contents in the leaves and bark from *C. zeylanicum* are 13 and 53 mg/kg, respectively. Significantly higher coumarin levels in *C. cassia, C. burmannii*, and *C. loureiroi* were reported, whereas *C. zeylanicum* contains only traces [47].

One of the possible side effects of cinnamaldehyde is hypersensitivity [48]. Although not so common, cases of intraoral allergic reactions due to different cinnamaldehyde-containing products have been reported in the literature [49,50]. Some evidence exists of the instability of *trans*-cinnamaldehyde when exposed to air or in blood vessels. A chemical reaction involving a transformation to cinnamic acid can occur, leading to reduction in the antibacterial activity [51]. Eugenol produces a possible irritation effect on the periapical tissues and could lead to necrosis of bone and cementum and to an alteration of the eruption of permanent teeth [52]. Reports of allergic contact dermatitis and stomatitis as a result of cinnamon EOs are rare [53,54], which could lead to orofacial granulomatosis [55]. In vivo studies reported a lack of significant side effects on the liver and kidneys, no hypersensitivity, and a wide therapeutic range of *C. zeylanicum* [56,57]. *C. zeylanicum* has a positive osteogenesis effect on pulp stem cells [58].

## 3. Antimicrobial Effect of Cinnamon EO and Cinnamon Extracts against Oral Pathogens

Cinnamon EO or cinnamon extracts could be used as antimicrobial agents. Steam distillation or organic solvents and alcohols are used for the preparation of the substances. The composition of the extracts or EO depends on the extraction method and protocol used [59].

The antimicrobial properties of cinnamon EO against different oral pathogens are subject to many researchers. These properties are assessed according to the minimum inhibitory concentration (MIC) values. Different degrees of antimicrobial activity of culinary herb EOs against the growth of cariogenic bacteria can be categorized into three groups: strong (MIC ≤ 0.1% (*v/v*)), moderate (MIC ≤ 1.0% (*v/v*)), and weak (MIC > 1.0% (*v/v*)) [7]. Natural products have strong antibacterial properties if their MIC is 500 µg/mL or less [60]. Hovijitra et al. categorized the efficacy of EO as strong (MIC < 0.5 µL/mL or 0.05% (*v/v*)), moderate (0.5 µL/mL ≤ MIC ≤ 1.0 µL/mL or 0.1% (*v/v*)), and weak (MIC >1.0 µL/mL) [61]. Other researchers used the following classification of antimycotic activity based on the MIC: <100 μg/mL (good), 100–500 μg/mL (moderate), 500–1000 μg/mL (weak), and >1000 μg/mL (inactive) [62].

### 3.1. Antimicrobial Effect against Caries Pathogens

Wiwattanarattanabut et al. reported good antimicrobial properties of cinnamon EO (*C. zeylanicum*) against two cariogenic bacteria: *S. mutans* KPSK and *Lactobacillus casei* [7]. Cinnamon EO exhibited a MIC and minimum bactericidal concentration (MBC) against *S. mutans* of 0.08% (*v/v*). Two-times higher MIC and MBC against *L. casei* were reported (0.16% (*v/v*)). The inhibition zone diameter was 32.17 ± 1.32 mm (±SD). In comparison, the inhibition zone of 0.2% chlorhexidine was 29.83 ± 0.75 mm (±SD). The inhibition zone of cinnamon EO against *L. casei* was 17.33 ± 1.03 mm. *S. mutans* biofilm mass was reduced by ≤35% and ≤19% after the treatment with 0.25% chlorhexidine for 24 h and 1 h, respectively. Cinnamon bark EO exhibited the strongest reductive effect, but the effect was not statistically significant (*p* > 0.05) against the *S. mutans* biofilm compared with all the tested herbal EOs. A 24-h exposure to cinnamon EO at its MIC, 2× MIC (0.16% (*v/v*)) and 4× MIC (0.32% (*v/v*)) reduced the biofilm mass by approximately 80%. Bacterial film reduction was also reported on implant surfaces [63].

Methanolic extract from *C. zeylanicum* produced inhibition zone diameters of 14.00 mm against *S. mutans* and 16.67 mm against *Lactobacillus acidophilus* [64]. The mean MIC (mg/mL) values were 13.44 against *S. mutans* and 5.18 against *L. acidophilus*. The mean MBC were 23.6 and 16.4 mg/mL, respectively. A combination of cinnamon and clove methanolic extracts resulted in larger inhibition zones. Ethanolic extract of *C. zeylanicum* showed good antimicrobial activity against *S. mutans* ATCC-700610: MIC = 195 µg/mL and MBC = 390 µg/mL [65].

In another study, nicotine-induced *S. mutans* UA159 biofilm formation proved sensitive to 2.5 mg/mL cinnamon water extract. Nicotine concentration-dependent reduction of the biofilm from 34% to 98% was reported (MIC and MBC were 2.5 mg/mL) [66].

*C. zeylanicum* leaves EO had a MIC of 250–500 µg/mL and MBC of 500–100 µg/mL against *S. mutans*, as reported by Galvão et al. [67].

The inhibitory effect of EO obtained from *C. cassia* bark added to a toothpaste against *S. mutans* was reported by Karadağlıoğlu et al. [68]. The combination of cinnamon EO and the selected commercially available herbal toothpastes resulted in a significant increase of the inhibition zone diameter (mean values between 36.33 and 39.67 mm).

Cinnamon EO showed the highest antibacterial activity against *S. mutans* among eight other EOs tested, including lime, spearmint, wintergreen, peppermint, lemongrass, cedarwood, clove, and eucalyptus EOs [69]. In a vast study investigating the antimicrobial properties of 32 EOs against oral pathogens, *S. mutans*, *Streptococcus sobrinus,* and *C. zeylanicum* EOs proved to be the most effective based on the inhibition zone measurements [70]. The antimicrobial effects of cinnamon against *S. mutans* and *Lactobacillus plantarum* proved to be stronger in comparison to tea tree, manuka, arnica, eucalyptus, and grapefruit [71]. In another study, the cinnamon EO did not prove its antibacterial properties against *S. mutans* [72]. The composition of the EO used in this study was mainly cinnamyl-alcohol (88.45%), cinnamyl-acetate (6.13%), and p-Eugenol (2.98%) One of the main antibacterial chemical compounds, cinnamaldehyde, was only responsible for 0.39% of the composition.

Data based on the MIC and MBC of cinnamon essential oil against caries pathogens are summarized in Table 2.

### 3.2. Antifungal Activity of Cinnamon EO and Cinnamon Extracts

The combined effect of cinnamon, clove, and oregano obtained by hydrodistillation against several *C. albicans* strains, *Candida tropicalis* IP 2148.93, and *Candida glabatra* DSM 11226 was reported by Brochot A. et al. [24]. The MIC and the minimal fungicidal concentration (MFC) varied between 0.01% and 0.05% *v/v*.

In another study [73], the antibacterial and antifungal effects of cinnamon EO, prepared by steam distillation against *S. mutans, S. aureus,* methicillin-resistant *Staphylococcus aureus (MRSA), C. albicans,* and *C. glabrata*, isolated from the oral samples, were assessed. The MIC of *C. zeylanicum* EO varied from 12.8 to 51.2 mg/mL (*S. mutans* was the most sensitive microorganism). The minimal inhibition of growth concentration of the tested microorganisms was 3.12% against *S. mutans* and 12.5% against *C. albicans* and *C. glabrata.*

The study of the comparative effects of cinnamon (*C. zeylanicum*), clove, and thyme EOs and nystatine on *C. albicans* growth resulted in similar inhibition zone diameters for cinnamon, clove, and nystatine [74].

*C. albicans* ATCC 28366 proved to be sensitive to cinnamon bark EO (*C. zeylanicum*) in another study [75]. The authors estimated MIC as ranging from 0.039% to 0.078%, with an MFC of 0.078%, which is a marker for the fungicidal properties of the EO. The inhibition of biofilm formation was obtained at 0.0049%. Cinnamon EO did not exhibit any effect on the *C. albicans* adhesion to oral epithelial cells.

The antifungal effects of the EO of *C. zeylanicum* leaves (eugenol content 68.96%) against several *Candida* spp., including reference strains and oral isolates of *C. albicans, C. tropicalis,* and *C. krusei,* showed a MIC from 62.5 to 1000 µg/mL and a MFC from 125 to 1000 µg/mL [76]. The MIC/MFC ratio was similar to that of nystatine. After exposure for 8 h, a significant reduction in the fungal growth was observed up to a concentration of 750 μg/mL. The suppression of biofilm formation at this concentration was also reported. Cinnamon EO was also able to reduce *Candida* spp. monospecies and multispecies in mature biofilm at 24 and 48 h. This concentration is safe to use, since no significant reduction in the viability of human peripheral blood mononuclear cells was observed with concentrations of up to 1000 μg/mL.

Another study [77] of the antifungal activity of *C. zeylanicum* leaf EO (eugenol content 82.30%) found that, at concentrations of 312.5 or 625.0 μg/mL, all 12 tested strains of *C. albicans* and *C. tropicalis* were inhibited. At the same concentrations, the authors observed a fungicidal effect of cinnamon EO against all 12 strains, except one (MFC was 312.5 or 625.0 μg/mL). The experimental mouth rinse, containing cinnamon EO at the MIC, used in this study did not significantly change the surface roughness or the Vickers microhardness of the heat-polymerized acrylic resin in contrast to the nystatine group. Even at low concentrations, *C. zeylanicum* EO has an inhibitory effect against *C. albicans*. The authors reported a MIC of 0.005% (0.05 µl/mL), an MFC of 0.01%, and a 90% reduction in bacterial biofilm at 0.039% [61]. A MIC <0.05 mg/mL for the methanolic extract of *C. zeylanicum* against *C. albicans* was reported in another study [78].

*C. zeylanicum* EO proved to be the most effective EO against 40 *C. albicans* isolates but to a lesser extent than the antifungal drugs and the mouthwashes [79]. A hydroalcoholic extract of *C. zeylanicum* was used to assess the antimycotic activity of cinnamon against fluconazole-resistant *C. albicans*. The MIC at a concentration of 15.62 μg/mL was reported [62]. The MIC and MFC at 65.5 μg/mL against *C. albicans* were reported by Cavalcanti et al. using *C. cassia* EO [80]. Khan et al. reported a MIC of 19.5 μg/mL and an MFC of 78 μg/mL against *C. albicans* ATCC-10231 [65].

Taguchi et al. studied the effects of *C. cassia, S. aromaticum,* and several other species and herbs against *C. albicans* in vitro and in an oral candidiasis murine model [81]. Cinnamon and clove hot water extracts inhibited the mycelial growth by 80% at concentrations of 1.0–5.0% and 5–25.0%, respectively. The original *C. cassia* preparation inhibited the mycelial growth by 80% at concentrations of 36.5–146 µg/mL. Only the 100% *C. cassia* preparation was able to improve the oral symptoms of candidiasis of the infected mice. Table 3 summarizes the data of the antifungal activity of cinnamon EO and cinnamon extracts.

### 3.3. Antimicrobial Effect against Endopathogens

Some studies focused on the antibacterial and antifungal effects of cinnamon EO and extracts against certain endodontic pathogens. The main cause of endodontic infection is the presence of microorganisms isolated as planktonic cells or biofilms [82]. *Enterococcus faecalis* is the most prevalent bacterium found in unsuccessful root canal treatments; some studies focused on the root canal irrigant efficiency against *C. albicans* [83,84].

The antimicrobial effect of steam-distillated *C. zeylanicum* bark EO against *E. faecalis* ATCC 29212 was reported by Abbaszadegan et al. [85], with a MIC at 0.01 mg/mL and MBC at 0.1 mg/mL. Cinnamon EO and the triple antibiotic paste used in the study were able to eliminate planktonic *E. faecalis* after 4 and 24 h, while calcium hydroxide paste failed to do so. Cinnamon EO showed better biocompatibility with experimental fibroblast cells in comparison to the other two substances.

The MIC of *C. zeylanicum* exhibited against planktonic *E. faecalis* was 10%, with complete bacterial inhibition after 30 s [86]. In a biofilm susceptibility assay on a cellulose nitrate membrane, complete bacterial inhibition was achieved after 12 h, in contrast to the faster activity of 3% sodium hypochlorite (NaOCl) of 2 min. The effect of cinnamon EO as a root canal irrigant is weaker than 3% NaOCl but could lead to 80–85% intracanal bacterial reduction [87]. A 20% ethanolic extract of *C. zeylanicum* was found to be even more effective against *E. faecalis* ATCC 29212 compared to 3% NaOCl [88]. The significant antimicrobial effect of *C. cassia* EO was proved against *C. albicans* and *E. faecalis* (MIC was 0.56 mg/mL). A wider inhibition zone in comparison to chlorhexidine digluconate (0.12%) and 1% sodium hypochlorite solutions was also observed [89]. Khan et al. reported MIC and MBC against *E. faecalis* ATCC-29212 at 95.7 and 1560 μg/mL, respectively [65].

### 3.4. Clinical Trials

In clinical trials and systematic reviews, data on the advantageous effects of EO-containing mouth rinses on gingival inflammation can be found [90,91]. Only one clinical trial was conducted on the effects of cinnamon in dentistry.

The plaque reductive and anti-inflammatory effects of cinnamon extract were studied in comparison to chlorhexidine mouthwash. Although chlorhexidine showed the maximum decrease in both plaque and gingival scores, the cinnamon extract’s effects were statistically insignificant [92].

### 3.5. Other Studies against Oral Pathogens

Cinnamon EO could be beneficial in halitosis treatment, since it reduced *Solobacterium moorei* biofilm formation, with MIC of 0.039% and MBC of 0.156% [93].

The antimicrobial effect of ethanolic extracts from *C. zeylanicum* and *Salvadora persica* against periodontal pathogens *P. gingivalis, Tannerella forsythia, Treponema denticola,* and *A. actinomycetemcomitans* was also studied. The results indicated the higher efficacy of cinnamon against all tested pathogens and the synergetic effect with antibiotics. The MIC ranged from 1.56 to 12.5 mg/mL and MBC from 6.25 to 75 mg/mL for different bacteria [94].

Bardají et al. investigated the antibacterial activity of *C. zeylanicum* EO against several oral pathogens. The MIC and MBC against *Fusobacterium nucleatum, Actinomyces naeslundii,* and *Prevotella nigrescens* were 125 μg/mL; the MIC and MBC against *S. mutans* were 200 and 400 μg/mL, respectively [95]. In contrast to many other studies, they did not detect cinnamaldehyde in the EO, and (*Z*)-isosafrole (85.3%) was the main component.

*C. zeylanicum* EO proved to be more effective in comparison to *C. zeylanicum* bark aqueous extract against *Staphylococcus auricularis, Acinetobacter lwoffii, C. albicans,* and *Micrococcus* species that were collected and isolated from volunteers’ oral cavities. *C. zeylanicum* EO was more effective than *S. aromaticum* EO, inhibiting the growth of all bacterial isolates [96]. This also indicates the activity of the experimental toothpaste used in the study containing: 35.00% calcium carbonate, 1.50% sodium lauryl sulfate, 30.00% glycerin, 1.00% sodium alginate, 00.12% sodium benzoate, 00.30% sodium saccharine, 2.50% plant extract, and purified water q.s.

Li et al. studied the antihalitosis effects of 40 Chinese herbs [97]. Fourteen herb extracts had volatile sulfur compound inhibition rates over 50%. The extract from *C. camphora* proved to be not so effective in halitosis treatment in vitro, with a volatile sulfur compound inhibition rate of only 25.29% and a 50.14% microorganism inhibition rate. Xu patented a method for the production of chewing gum for the prevention and cure of decayed tooth and periodontitis with one of the active ingredients being *Cinnamomum japonese* extract [98].

Cinnamon EO could be useful in antibiotic treatments due to the possible synergetic effect. The combination of *C. zeylanicum* EO with amikacin showed a significant synergetic effect against *Acinetobacter* species with reduction of the MIC of amikacin [99]. The possible use of cinnamon bark EO as a modifying agent in the treatment of antibiotic-resistant bacteria was reported in combination with piperacillin [100].

## 4. Main Constituents of *Cinnamomum* spp. and Their Antibacterial Properties against Oral Pathogens

### 4.1. Cinnamaldehyde

*Trans*-Cinnamaldehyde (*t*-Cinnamaldehyde or (*E*)-Cinnamaldehyde) was reported to be the main constituent of cinnamon EO and extracts. This molecule provides a major contribution to cinnamon’s organoleptic and antibacterial properties.

Wang et al. studied the antibacterial effect of *C. zeylanicum* bark EO and its main constituent (57.97% cinnamaldehyde) against *P. gingivalis* [101]. The authors confirmed that cinnamaldehyde is the constituent responsible for the antibacterial properties of *C. zeylanicum*, as it shows strong antibacterial effects; the MIC of the EO was 6.25 μg/mL, and the MIC of cinnamaldehyde was 2.5 μM. The inhibitions of the *P. gingivalis* biofilm by 74.5% and 67.3%, respectively, were also observed.

A study of the antimicrobial properties of the main constituents of *C. zeylanicum* EO showed that cinnamaldehyde was the most active against the two oral pathogens (*S. mutans* and *Streptococcus sobrinus*), with inhibition zones ranging from 4.2 to 5.7 cm. Eugenol and cinnamyl alcohol showed more effective activity against *S. sobrinus* in comparison to *S. mutans*. Both tested strains showed moderate sensitivity to 3-carene, α-Terpinene, benzaldehyde, linalool, β-Caryophyllene, α-Humulene, α-Terpineol, hydrocinnamic aldehyde, hydrocinnamyl acetate, and cinnamyl acetate. The MIC of cinnamaldehyde against both bacterial strains was 0.02% (*v/v*), whereas the MBCs against *S. mutans* and *S. sorbinus* were 0.2% and 0.1% (*v/v*), respectively [70]. *C. zeylanicum*, due to its main constituent *t*-Cinnamaldehyde, showed the highest antibacterial activity against several bacteria, with clinical significance among all the tested EOs (cumin (*Cuminum cyminum*), *C. verum*, cardamom (*Amomum subulatum*), and clove (*S. aromaticum*)) [102]. In another study, the antimicrobial activity of cinnamaldehyde against *Pseudomonas aeruginosa* proved to be stronger than the antimicrobial activity of eugenol [103]. In a study by Pei, the MICs of cinnamaldehyde, thymol, and carvacrol alone against *Escherichia coli* were found to be 400 mg/mL, whereas the MIC of eugenol was four times higher at 1600 mg/mL [104].

Cinnamaldehyde proved to have strong antifungal activity against the standard *C. albicans* strain and 26 oral isolates of *C. albicans* [105]. The in vitro antifungal effect against fluconazole-resistant *C. albicans* isolates (MIC 100–500 μg/mL; 0.01–0.05%) was also reported [106]. Taguchi et al. studied the antifungal activity of cinnamaldehyde and coumarin as the main compounds in a *C. cassia* preparation. A mycelial growth inhibition of 80% was achieved with 8.19–20.5 µg/mL of cinnamaldehyde and 128–320 µg/mL of coumarin, which indicated that cinnamaldehyde has superior activity to that of coumarin. [81]. Firmino et al. compared the abilities of *C. zeylanicum* EO, *C. cassia* EO, and (*E*)-Cinnamaldehyde to reduce the biofilm biomass by 100% [32]. *C. zeylanicum* EO proved to be the most effective against *E. coli, P. aeruginosa*, and *Streptococcus pyogenes*. The three tested substances reduced the *S. aureus* biofilm at an equal concentration, 0.25%, and only against *Staphylococcus epidermidis* did (*E*)-Cinnamaldehyde show better activity (the minimal concentration was two times lower than that of the two EOs tested). This means that the effect of the EO or its main constituent depends on the bacterial strain and the possible synergetic effects with the other constituents in the EOs.

### 4.2. Eugenol

Eugenol is another powerful substance present in cinnamon EO, although it is commonly associated with clove [107]. Since it is one of the most abundant compounds in cinnamon EO and extracts reported by some authors, its strong antimicrobial properties may play a significant role in oral health.

The antimicrobial effect of eugenol against different oral pathogens and several modes of action have been reported. Eugenol showed a strong antibacterial effect against *P. gingivalis* ATCC 33277, with a MIC and MBC of 31.25 and 125 μM, respectively [108]. It destroyed the cell membrane in a dose-dependent manner, leading to cell shrinkage and death. Eugenol also reduced the biofilm formation of *P. gingivalis* and showed an ability to reduce the existing biofilm. A significant decrease in the expression of six virulence factor genes was also reported. A significant antibiofilm activity of eugenol against *C. albicans* and *S. mutans* was also observed in single and mixed biofilms [109]. Another study on the suppression effects of eugenol on biofilm- and quorum-sensing-related genes of *S. mutans* showed the significant effects of this compound at the sub-MIC [110]. The acid production and the synthesis of water-insoluble glucans by *S. mutans* were suppressed by eugenol. Eugenol also significantly inhibited the adherence of *S. mutans* to saliva-coated hydroxylapatite beads, and the effect was concentration-dependent. Eugenol treatment reduced the scores of smooth surfaces and sulcal caries in rats [111].

A comparative study of the antimicrobial effects of eugenol, β-Caryophyllene, and clove oil against several oral bacteria (*S. mutans*, *Streptococcus sanguinis*, *S. sobrinus*, *Streptococcus ratti*, *Streptococcus criceti*, *Streptococcus anginosus*, *Streptococcus gordonii*, *A. actinomycetemcomitans*, *F. nucleatum*, *P. intermedia*, and *P. gingivalis*) was conducted [112]. The MIC of eugenol and β-Caryophyllene varied from 0.1 to 0.8 mg/mL and from 0.8 to 12.8 mg/mL, respectively. The MBC of eugenol was lower than that of β-Caryophyllene (0.1–0.8 and 1.6–12.8 mg/mL, respectively). Eugenol showed a slightly stronger antibacterial activity in comparison to clove oil against some of the microorganisms. A synergistic effect with gentamicin and ampicillin was observed.

Eugenol nanoemulsion gel exhibited anti-inflammatory activity and analgesic and antibacterial effects, showing promise for the treatment of periodontal disease [113]. The antibacterial effect of zinc oxide eugenol paste against *E. faecalis* was confirmed [114].

### 4.3. Linalool

Park et al. studied the antibacterial properties of linalool and α-Terpineol against several periodontopathogens and caries pathogens, including different strains from *P. gingivalis, P. intermedia, Prevotela nidrescens, F. nucleatum, Aggregatibacter actinomycetemcomitans, S. mutans*, and *S. sobrinus* [115]. The MIC of linalool against periodontal pathogens varied between 0.1 and 1.6 mg/mL; with the exception of four out of 15 strains, the MIC was 0.1 or 0.2 mg/mL. The MBC for all tested strains, except for two, was between 0.1 and 0.8 mg/mL. One strain of *P. gingivalis* and one of *P. nigrescens* had an MBC of 1.6 mg/mL. Linalool showed lower antibacterial activity against cariogenic bacteria, as the MIC and MBC ranged from 0.1 to 3.2 mg/mL. Most of the 16 strains tested showed MIC and MBC values of 0.4 mg/mL and above, except for one strain of *S. mutans* (0.1 mg/mL). α-Terpineol showed MIC and MBC values against all tested periodontopathogens between 0.1 and 0.8 mg/mL. The antimicrobial effect against cariogenic bacteria was slightly weaker, since the MIC and MBC varied between 0.4 and 1.6 mg/mL, with the exception of the most sensitive against linalool, the *S. mutans* strain (MIC and MBC were 0.1 mg/mL). Linalool and α-Terpineol showed increased toxicity on the KB cell line at concentrations of 0.4 mg/mL and above, which should be their maximum concentration if used in toothpastes or mouthwashes.

### 4.4. β-Caryophyllene

The efficacy of β-Caryophyllene against *S. mutans* was reported [116]. The inhibitory effect of β-Caryophyllene on the growth of *S. mutans* started at concentrations above 0.078%, and complete inhibition was observed at 0.32% and above. *S. mutans* biofilm formation was suppressed by 0.16% β-Caryophyllene. In a mature *S. mutans* biofilm, 1.25% β-Caryophyllene exhibited disruptive and bactericidal activity. At a 0.039% concentration, β-Caryophyllene significantly inhibited the expression of GtfB and GtfC and reduced the expression of GtfD, pointing to the mechanism of antibacterial action.

The antibacterial activity of β-Caryophyllene against three periodontal pathogens, *P. gingivalis, T. forsythia*, and *T. denticola*, was also studied [117]. *T. forsythia* and *P. gingivalis* growth inhibitions were significant at concentrations of 0.019% and 0.004% and above, respectively. β-Caryophyllene interfered with lipopolysaccharide binding to CD14 or LBP, which resulted in inhibition of the induction of cytokine expression by lipopolysaccharides. A positive effect in volatile sulfur compounds reduction was also observed; thus, β-Caryophyllene could reduce halitosis.

## 5. Discussion

The review focused on the implications for cinnamon EO and extracts, as well as isolated bioactive compounds used in dental medicine. Since bacterial and fungal infections are the main causes of the most common dental diseases, the effects of cinnamon EO, cinnamon extracts, and the main compounds in treatments mainly depend on their antimicrobial and anti-inflammatory properties.

Few studies assessed the antibacterial effects of cinnamon EO and extracts against cariogenic bacteria, and approximately a dozen studies focused on the antifungal effect. Cinnamon has fungicidal and bactericidal effects based on the MIC, MBC, and MFC data. Wide ranges of MIC, MBC, and MFC have been reported, probably because different material sources and extraction methods were used. The mean values were calculated and are presented in Table 4 as percentages. The antifungal activity of cinnamon is more pronounced compared to its antibacterial properties, given the lower values of the MIC and MFC reported. Thus, cinnamon EO and cinnamon extracts could be useful in candidiasis treatments as the main or a complementary agent.

Based upon these data, we conclude that cinnamon EO has strong antimicrobial effect against *Candida* spp. and moderate-to-weak effects against caries pathogens [7,60,61,62]. Still, the number of studies is low.

Cinnamon EO and extracts showed strong effects against endodontic pathogens. Thus, in certain cases, they can be used as an irrigant or as a temporary medicine on impregnated cotton pellet between clinical procedures. This would be appropriate in cases of a sodium hypochlorite allergy. They can also be added to zinc oxide formulations. Eugenol has already proved its antibacterial properties in dental medicine and has been used for centuries. Cinnamaldehyde, conversely, is not well-studied in endodontics. Further research on the positive effects of cinnamaldehyde-based medicines is needed.

To the best of our knowledge, only one clinical trial on the plaque reductive and anti-inflammatory effects of cinnamon extract in comparison to chlorhexidine mouthwash is described in the literature. Most of the other studies examined the antimicrobial effects of cinnamon and its main constituents in vitro. More in vivo studies would provide useful clinical information. Based on the antimicrobial properties, it seems reasonable to include cinnamon EO, cinnamon extracts, or major constituents in mouthwashes, toothpastes, and denture-cleansing solutions. Some authors proposed herbal dental toothpaste formulations that showed positive effects on plaque control, gingivitis, and halitosis [118,119]. One possible herbal dentifrice formulation was prepared by mixing 1 g of NaHCO_3_, two to three drops of essential peppermint oil, one drop clove of oil, 1 mL of coconut oil, and four to five drops of water [119]. Clove oil could be substituted with cinnamon EO.

Pure cinnamon compounds show different antimicrobial properties. The reported MIC of cinnamaldehyde against *S. mutans* and *S. sorbinus* was 0.02% (*v/v*), and the MBC was 0.2% and 0.1% (*v/v*), respectively, which is in the range of the MIC against *C. albicans* (0.01–0.05%) but is higher than the mean MIC of the cinnamon EO and extracts (Table 4). This suggests that cinnamaldehyde could be used as an alternative to cinnamon EO [32,70,106]. The antimicrobial effects of cinnamaldehyde and eugenol against *P. gingivalis* produced MICs of 2.5 and 31.25 μM, respectively [101,108]. The contribution of cinnamaldehyde to cinnamon’s antimicrobial properties was reported in other studies [81,103]. Eugenol showed greater antibacterial activity against several oral pathogens in comparison to β-Caryophyllene, although the latter proved to be effective against *S. mutans* and even more efficient against *P. gingivalis*, *Tannerella forsythia*, and *Treponema denticola* [113,118,119]. Linalool and α-Terpineol also proved effective against several periodontopathogens and caries pathogens, but their concentrations in toothpastes or mouthwashes should not exceed 0.04% [115].

Cinnamon EO, cinnamon extracts, and pure compounds exhibit significant antimicrobial properties; the choice of which in dental practice depends on different factors. EOs are natural and reachable solutions, but their properties depend on many factors, and their active ingredient contents are not constant [120,121]. There are seasonal changes in the contents, and the chemical compositions may vary considerably between species [122]. Thus, from a pharmaceutical point of view, if precise active ingredient concentrations is the goal, using pure compounds like cinnamaldehyde and eugenol seem to be the best alternatives. These substances could be included in certain formulations by dental material manufacturers. Since the effects of the EO depend on the interaction of all its ingredients, not only its main components, further investigations should be conducted to determine the synergetic effects between the pure compounds [123,124]. The compositions of the cinnamon extracts and their properties depend on the method used [59].

What cinnamon species or part of the plant should be used is another question that remains unanswered. Species high in cinnamaldehyde and eugenol and low in coumarin are preferable. A combination of leaf and bark EOs, due to the high cinnamaldehyde and eugenol contents, would contribute to the effective antimicrobial properties of the product. The studying and selection of certain chemo types seems reasonable. *C. zeylanicum* is one of the most-studied cinnamon species; it is generally safe to use and has shown good antimicrobial properties.

## 6. Conclusions

Cinnamon EO, cinnamon extracts, and the main components show significant antimicrobial activities against oral pathogens and could be beneficial in caries and periodontal disease prevention, endodontics, and candidiasis treatment. Further research focused on the clinical use of cinnamon EO-containing mouth care products should be conducted.

## Figures and Tables

**Table 1:** Most abundant compounds found in different cinnamon species and parts of the plant.

Cinnamon Species	Part of the Plant	Essential Oil (EO) or Extract	Main Compounds	Reference
*C. zeylanicum*	flower	EO	(*E*)-Cinnamyl acetate (41.98%), *trans*-alpha-Bergamotene (7.97%), caryophyllene oxide (7.2%)	[27]
*C. zeylanicum*	leaf	-	Eugenol (87.3%)	[28]
*C. zeylanicum*	bark	-	(*E*)-Cinnamaldehyde (49.9%)	[28]
*C. zeylanicum*	fruit	EO	*trans*-Cinnamyl acetate and β-Caryophyllene	[29]
*C. zeylanicum*	bud	EO	α-Bergamotene and α-Copaene	[30]
*C. cassia*	bark	EO	*t*-Cinnamaldehyde (75–97%)	[31]
*C. cassia*	leaf	EO	*t*-Cinnamaldehyde (33.5–69.3%) and methoxy cinnamaldehyde (11.29–23.37%)	[31]
*C. zeylanicum*	bark	EO	(*E*)-Cinnamaldehyde (68.7%), Cinnamyl (*E*)-acetate (7.12%), and eugenol (6.33%)	[32]
*C. cassia*	bark	EO	(*E*)-Cinnamaldehyde (90.22%)	[32]
*C. burmanni*	leaf	extract	d-borneol	[33]
*C. burmanni*	stick	extract	(*E*)-Cinnamaldehyde and polyphenols	[34]
*C. burmanni*	leaf and bark	EO	*trans*-Cinnamaldehyde (68.30–84.71%), cinnamyl acetate (2.97–16.10%), cinnamyl alcohol, and *trans*-Cinnamic acid	[35]
*C. burmanni*	bark	extract	*trans*-Cinnamaldehyde	[36]
*C. burmanni*	bark	extract	*trans*-Cinnamaldehyde, coumarin, and brazilin	[37]
*C. camphora*	leaf	-	Isoborneol-type, camphora-type, cineole-type, linalool-type, and borneol-type	[38]
*C. tamala*	leaf	-	Eugenol	[39]
*C. osmophloeum*			α-Pinene, camphene, benzaldehyde, β-Pinene, 3-pheayl pionaldehyde, *cis*-Cinnamaldehyde, *trans*-Cinnamaldehyde, isobornylacetate, eugenol, and cinnamyl acetate	[40]
*C. osmophloeum*	leaf	EO	Linalool, *trans*-Cinnamyl acetate, camphor, cinnamaldehyde	[41]
*Cinnamomum altissimum*	bark	EO	Linalool (36.0%), methyl eugenol (12.8%), limonene (8.3%), α-Terpineol (7.8%), and terpinen-4-ol (6.4%)	[42]
*C. cassia*	Bark	extract	(*E*)-Cinnamaldehyde (62.96%), coumarin (11.36%), α-Copaene (3.78%), 3-methoxy-1,2-propanediol (3.26%), and α-Cuaiene (3.19%)	[43]
*C. loureiroi*	bark	extract	(*E*)-Cinnamaldehyde (51.69%), α-Copaene (16.14%), cinnamaldehyde dimethyl acetal (5.66%), β-Cadinene (3.19%), and α-Muurolene (4.78%).	[43]
*C. burmannii*	bark	extract	(*E*)-Cinnamaldehyde (34.44%), eugenol (25.67%), coumarin (16.82%), borneol (3.28%), and methyl cinnamate (3.16%)	[43]
*C. wilsonii*	bark	extract	Linalool (23.66%), (*E*)-Cinnamaldehyde (19.63%), citral (15.45%), (*E*)-Cinnamyl acetate (8.65%), and 1,8-Cineole (5.54%)	[43]
*C. verum*	leaf	-	Eugenol (85.66%), acetyl eugenol (6.07%), cinnamaldehyde, β-Caryophyllene (1.08%)	[44]
*C. verum*	bark	-	Cinnamaldehyde (67.57%), eugenol (16.03%), α-Pinene (5.76%), linalool (3.78%), β-Caryophyllene (3.66%)	[44]
*C. dubium*	leaf	-	Geraniol (24.05%), cinnamyl alcohol (15.65%), eugenol (9.17%), β-Caryophyllene (5.60%), and α-Pinene (4.04%)	[44]
*C. dubium*	bark	-	β-Caryophyllene (41.31%), cinnamyl alcohol (8.61%), hydro cinnamic aldehyde (7.70%), eugenol (5.08%), and garaniol (3.86%)	[44]
*C. rivolorum*	leaf	-	Eugenol (63.45%), α-Pinene (3.17%), geraniol (2.06%), cinnamaldehyde (1.57%), and β-Caryophyllene (1.24%)	[44]
*C. rivolorum*	bark	-	Cinnamaldehyde (31.78%), eugenol (22.29%), β-Caryophyllene (8.21%), and geraniol (7.76%)	[44]
*C. sinharajense*	leaf	-	Eugenol (87.53%), cinnamaldehyde (2.04%), cinnamyl alcohol (1.50%), and β-Caryophyllene (1.04%)	[44]
*C. sinharajense*	bark	-	Cinnamaldehyde (57.46%), cinnamyl acetate (13.69%), and β-Caryophyllene (4.54%)	[44]
*C. citriodorum*	leaf	-	Linalool (30.71%), cinnamyl alcohol (5.36%), cinnamyl acetate (3.20%), citronellol (2.44%), and cinnamaldehyde (2.27%)	[44]
*C. citriodorum*	bark	-	Cinnamaldehyde (42.74%), geraniol (19.95%), linalool (8.94%), eugenol (4.0%), and β-Caryophyllene (3.56%)	[44]

**Table 2:** Minimal inhibitory concentration (MIC) and minimal bactericidal concentration (MBC) of cinnamon EO and cinnamon extracts against cariogenic bacteria.

Cinnamon Species	EO or Extract	Bacterial Strains	MIC (%)	MBC (%)	Reference
*C. zeylanicum*	EO	*S. mutans* *L. casei*	0.08 0.16	0.08 0.16	[7]
*C. zeylanicum*	Methanolic extract	*S. mutans* *L. acidophilus*	1.34 0.52	2.36 1.64	[64]
*C. zeylanicum*	Ethanolic extract	*S. mutans*	0.02	0.04	[65]
*C. zeylanicum*	Water extract	*S. mutans*	0.25	0.25	[66]
*C. zeylanicum*	EO	*S. mutans*	0.02–0.05	0.05–0.1	[67]

**Table 3:** Minimal inhibitory concentration (MIC) and minimal fungicidal concentration (MFC) of cinnamon EO and cinnamon extracts.

Cinnamon Species	EO or Extract	Candida Species	MIC (%)	MFC (%)	Reference
*C. zeylanicum*	EO	*C. albicans* *C. tropicalis* *C. glabatra*	0.01–0.05	0.01–0.05	[24]
*C. zeylanicum*	EO	*C. albicans* *C. glabatra*	5.12	-	[73]
*C. zeylanicum*	EO	*C. albicans*	0.039–0.078	0.078	[75]
*C. zeylanicum*	EO	*C. albicans,* *C. tropicalis* *C. krusei*	0.006–0.1	0.01–0.1	[76]
*C. zeylanicum*	EO	*C. albicans,* *C. tropicalis*	0.03–0.06	0.03–0.06	[77]
*C. zeylanicum*	EO	*C. albicans*	0.005	0.01	[61]
*C. zeylanicum*	Methanolic extract	*C. albicans*	0.005	-	[78]
*C. zeylanicum*	Hydroalcoholic extract	*C. albicans*	0.001	-	[62]
*C. cassia*	EO	*C. albicans*	0.006	0.006	[80]
*C. zeylanicum*	Ethanolic extract	*C. albicans*	0.002	0.008	[65]

**Table 4:** The MIC, MBC, and MFC (%) of cinnamon EO against oral pathogens (mean and standard deviation (SD)).

	MIC		MBC		MFC	
	Mean	SD	Mean	SD	Mean	SD
Caries pathogens	0.31	0.45	0.585	0.896	-	-
*Candida* spp.	0.030	0.032	-	-	0.036	0.032
